# Case report: A case of severe capecitabine toxicity due to confirmed in trans compound heterozygosity of a common and rare DPYD variant

**DOI:** 10.3389/fphar.2024.1459565

**Published:** 2024-09-23

**Authors:** Amy de Haar-Holleman, Pieter-Jan Cortoos, Jelle Vlaeminck, Paulien Van Landuyt, Stephane Steurbaut, Freya Vaeyens, Vincent Haufroid

**Affiliations:** ^1^ Department of Medical Oncology, Universitair Ziekenhuis Brussel (UZBrussel), Brussels, Belgium; ^2^ Translational Oncology Research Center (TORC), Vrije Universiteit Brussel (VUB), Brussels, Belgium; ^3^ Pharmacy Department, Universitair Ziekenhuis Brussel (UZBrussel), Brussels, Belgium; ^4^ Faculty of Medicine & Pharmacy, Vrije Universiteit Brussel (VUB), Brussels, Belgium; ^5^ Centre for Medical Genetics, Research Group Genetics, Reproduction and Development, Clinical Sciences, Vrije Universiteit Brussel (VUB), Universitair Ziekenhuis Brussel (UZ Brussel), Brussels, Belgium; ^6^ Louvain Center for Toxicology and Applied Pharmacology, Institut de Recherche Expérimentale et Clinique (IREC), Université Catholique de Louvain (UCLouvain), Brussels, Belgium; ^7^ Department of Clinical Chemistry, Cliniques Universitaires Saint-Luc, Brussels, Belgium

**Keywords:** case report, capecitabine, DPYD gene polymorphism, pheno- and genotyping, rare variant

## Abstract

Variations in the activity of the enzyme dihydropyrimidine dehydrogenase (DPD) are associated with toxicity to fluoropyrimidine-containing chemotherapy. Testing of DPD deficiency either by targeted genotyping of the corresponding *DPYD* gene or by quantification of plasma concentration of uracil and dihydrouracil (phenotyping approach) are the two main methods capable of predicting reduced enzymatic activity in order to reduce adverse reactions after fluoropyrimidine treatment. In this paper, we describe a patient with locally advanced colon carcinoma with severe toxicity following capecitabine therapy. Whereas targeted genotyping for the 4 most common *DPYD* variants analysis revealed heterozygous presence of the c.2846A>T variant, which is a relatively common variant associated with a partial deficiency, additional phenotyping was compatible with a complete DPD deficiency. Subsequent sequencing of the whole *DPYD* gene revealed the additional presence of the rare c.2872A>G variant, which is associated with a total loss of DPD activity. A clinical case of *in trans* compound heterozygosity of a common and a rare *DPYD* variant (c.2846A>T and c.2872A>G) has, to the best of our knowledge, not been previously described. Our case report shows the importance of performing either preemptive phenotyping or preemptive complete genetic analysis of the *DPYD* gene for patients planned for systemic fluoropyrimidines to identify rare and low frequency variants responsible for potentially life-threatening toxic reactions.

## Introduction

Fluoropyrimidines belong to the class of antimetabolite drugs that form an integral backbone in the treatment of patients with cancers arising from the gastrointestinal tract, breast, head and neck. This includes 5-fluorouracil (5-FU) and the oral prodrugs capecitabine and tegafur. Conversion of 5-FU to dihydrofluorouracil is the first and rate-limiting step of the 5-FU degradation pathway and is regulated by the dihydropyrimidine dehydrogenase (DPD) enzyme, encoded by the *DPYD* gene (Etienne-Grimaldi et al., 2023). This enzyme converts up to 85% of 5-FU to inactive metabolites. Additionally, approximately 5% is excreted in urine. Deficiencies in DPD enzyme activity leads to increased intracellular concentrations of the active metabolites of 5-FU. This can lead to severe toxicity (neutropenia, mucositis and diarrhea), which is fatal in approximately 1% of patients. Because DPD deficiency affects 5%–7% of the Caucasian population, the European Medicines Agency (EMA) recommended in 2020 that prior to commencing fluoropyrimidine-containing therapy, patients should be tested either by genotyping the corresponding *DPYD* gene, or by phenotyping which is done by measuring plasma uracil concentrations or the dihydrouracil:uracil (UH2:U) ratio (Etienne-Grimaldi et al., 2023; European Medicines Agency, 2020). Both tests have their strengths and limitations, and no current recommendations are available regarding the preferred type of testing.

In this case report, we present a patient with severe 5-FU related toxicity, which was caused by a combination of a common *DPYD* variant with decreased DPD activity, and an initially undetected and uncommon *DPYD* variant associated with absent DPD activity. This case illustrates the added value of conducting a *DPD* phenotyping test and the potential danger of restricted testing of the locally most predominant *DPYD* variants. The CARE checklist was used when writing this report (Gagnier et al., 2013).

## Case description

**FIGURE 1:**
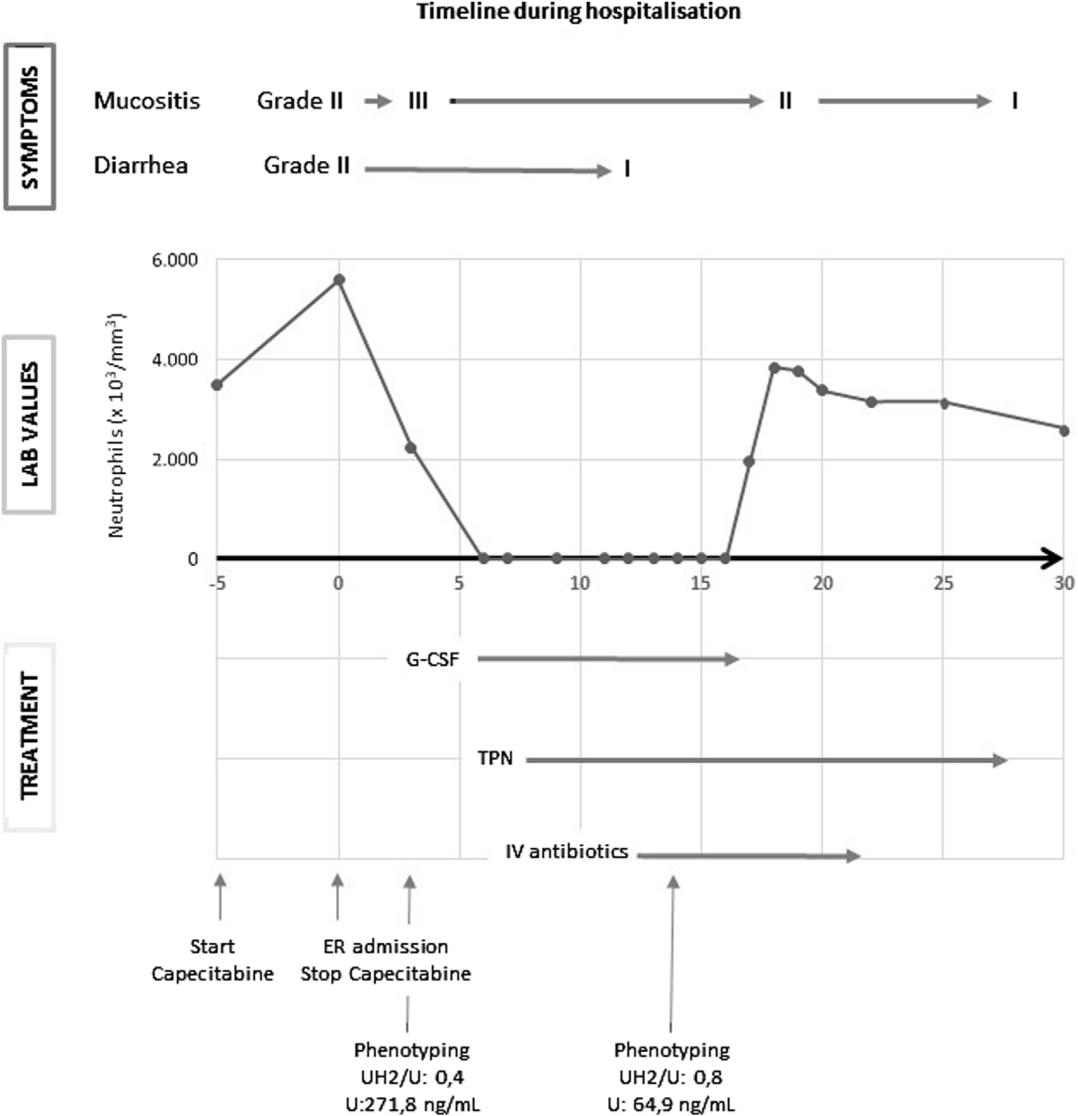
G-CSF: granulocyte colony-stimulating factor; IV: intravenous; TPN: total parenteral nutrition; U: uracil; UH2: dihydrouracil.

A 56-year-old Caucasian male with no comorbidity was diagnosed in another hospital with a well-differentiated adenocarcinoma of the distal sigmoid after a positive fecal occult blood test (FOBT). Imaging showed no evidence of distant metastases. He underwent a laparoscopic rectosigmoidectomy plus a partial mesorectal excision (PME) in October 2023 with the tumor being staged as pT4aN1M0. He decided to transfer to our hospital for adjuvant chemotherapy. As per international guidelines, he started adjuvant therapy with CAPOX (oxaliplatin 130 mg/mg^2^ day 1, capecitabine 1,000 mg/mg^2^ for 14 days, every 3 weeks). Upon diagnosis, targeted *DPYD* genotyping had already been performed in the referring hospital for the most common variants, i.e., DPYD*2A, DPYD*13, HapB3 and c.2846A>T, and showed the patient was heterozygous for the c.2846A>T variant (p.D949V, rs67376798). This variant is well known and has been functionally characterized as conferring a decreased function to the DPYD enzyme (Offer et al., 2014) and is included in the 2017 update of the Clinical Pharmacogenetics Implementation Consortium (CPIC) guideline, the 2019 Dutch Pharmacogenetics Working Group (DPWG) guideline for fluoropyrimidine dosing and the PharmGKB database as a tier I variant (Amstutz et al., 2018; Lunenburg et al., 2020). As genotyping results were already available and further delay of adjuvant therapy was deemed undesirable, an additional phenotyping test that is normal practice in our hospital was not performed in this case. After consultation with the clinical pharmacist and in accordance with the guidelines, the first cycle was started with 50% dose reduction of capecitabine, with the caveat to monitor for possible additional toxicity in case of rare undetected variants. A treatment timeline is presented in Figure 1. After 5 days of capecitabine treatment, he presented to the emergency department with vomiting and diarrhea. Clinical examination was remarkable for facial erythema and stomatitis. He was admitted for rehydration and capecitabine was discontinued. Despite the use of supportive therapy, his symptoms progressively aggravated.

Suspecting 5-FU-related toxicity, DPD phenotyping was performed. Quantification of uracil (U) and dihydrouracil (UH2) was performed by liquid chromatography with tandem mass spectrometry after protein precipitation followed by liquid-liquid extraction using a fully validated method certified according to ISO-15189 standards, with additional external quality control organized by Asqualab, France (ISO-9001 certified EQC programme) to ensure accuracy of the results. According to literature, in particular Belgian recommendations, a reference value of >14 ng/mL for pre-treatment uracilemia was used to detect DPD deficiency, provided that pre-analytical conditions have been respected (plasma freezing time less than 1h30) and the patient’s renal function has been taken into account (eGFR >60 mL/min) (Casneuf et al., 2022). Thresholds for differentiating between partial and total DPD deficiency are less clear, although a value of 100 ng/mL has been proposed in the Belgian guidelines. A UH2/U ratio value of <1 is usually proposed to establish a diagnosis of total DPD deficiency, whereas the value of this ratio is usually >10 in patients with normal DPD activity. A first phenotyping test was sent on day 2 of hospitalization but was reported as non-interpretable (uracil (U): 271.8 ng/mL, dihydrouracil (UH2): 116.8 ng/mL; UH2/U: 0.4) due to the detected presence of 5-FU artificially increasing uracilemia (Thomas et al., 2021). On day 2 of hospitalization, he developed transfusion-dependent pancytopenia, which deepened over the next days. Despite the administration of granulocyte colony-stimulating factor (G-CSF), the nadir for neutrophil count was at 0,00 ×10^3^/µL on day 10 of hospitalization, and for thrombocytes 10 × 10^3^/µL on day 7. On day 5 of hospitalization total parental nutrition (TPN) was initiated due to severe mucositis. On day 5 of hospitalization, he developed neutropenic fever, which was empirically treated with piperacillin/tazobactam and vancomycin. *Staphylococcus aureus* was cultured from his sputum, upon which vancomycin was switched to flucloxacillin. Due to clinical improvement and recovery of cytopenia, flucloxacillin was discontinued on day 15 of hospitalization and piperacillin/tazobactam on day 21. From day 31 of hospitalization his TPN was gradually diminished due to increased oral intake. On day 28 of hospitalization a second phenotyping test (at a distance from capecitabine administration and interference by competitive inhibition of the DPD enzyme) was reported to be compatible with a complete DPD deficiency, which was in line with the clinical situation of our patient (U: 64.9 ng/mL; UH2: 54.4 ng/mL; UH2/U: 0.8). Our patient was finally discharged on day 34. After extensive counseling he decided not to restart further adjuvant treatment and is now closely clinically monitored with regular scans. At the time of writing, he is still in remission.

**FIGURE 2:**
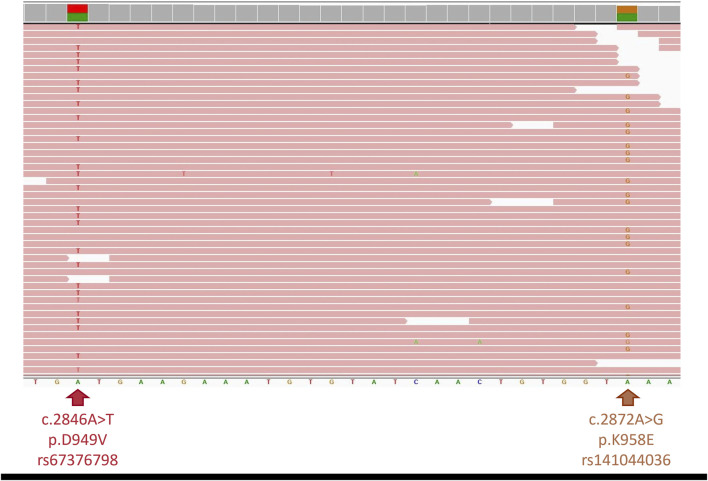
Integrative Genomics Viewer screenshot of genomic region. Next-generation sequencing showing both *DPYD* variants (c.2846A>T (left) and c.2872A>G (right)) present in different sequence reads, confirming *in trans* phasing.

Considering his extreme toxicity despite a 50% dose reduction of capecitabine and a complete DPD deficiency according the second phenotyping test, DPYD full sequencing was carried out (using SOPHiA DDM^®^ for Pharmacogenomics on a MiSeq Illumina Inc., United States) confirming the additional heterozygous presence of a NM_000110.4:c.2872A>G (p.K958E, rs141044036) variant, known to be associated with a total loss of DPD activity (Offer et al., 2014). The identification of two *DPYD* variants combined with the observed severe toxicity suggested the patient was an *in trans* compound heterozygous carrier. To confirm this hypothesis, next-generation sequencing using a pan-cancer capture based 380 gene panel, was performed on a NovaSeq 6000 (Illumina Inc., United States). Sequencing of the entire *DPYD* gene confirmed the presence of both, already identified, variants. Read visualisation at the level of their genomic locations (c.2846A>T: g.97547947; c.2872A>G: g.97547921) showed they were present in separate reads (variant allele frequencies: 50% and 48% respectively), confirming the *in trans* compound heterozygous state of the patient (Figure 2). This *in trans* compound heterozygous state of two not fully functional *DPYD* alleles (c.2846A>T/c.2872A>G) corresponds to a DPD activity score of 0.5 and therefore a poor metabolizer matched phenotype as per CPIC guideline (Amstutz et al., 2018).

## Discussion

This case report highlights the clinical importance of screening for DPD deficiency prior to initiating fluoropyrimidine therapy using either targeted genotyping or phenotyping by measuring pre-treatment uracilemia. Although not yet recommended in the United States by the Food and Drug Administration (FDA), this screening strategy is currently strongly recommended in Europe by EMA since 2020 (European Medicines Agency, 2020; de With et al., 2023) and without it, our patient would have received a standard dose of 5-FU and would most likely not have survived his treatment.

The question arises which method forms the optimal strategy for preemptive DPD screening. The phenotyping test would have been able to predict a major risk of toxicity in our patient whereas the used targeted genotyping test failed to do, as the additional rare variant was not represented. The phenotyping test, however, has drawbacks too. Its main constraint is that it requires plasma to have been separated and frozen within 90 min of blood collection (Maillard et al., 2023; Thomas et al., 2023). Furthermore, knowledge of the patient’s glomerular filtration rate (eGFR) is necessary, as an increase in U and UH2 is observed in case of renal failure, without being linked to DPD deficiency. In this case UH2/U ratio remains in the normal range as both components are increased, still allowing a reliable interpretation of results (Narjoz et al., 2023). In addition, there is a between-subject variability and possible circadian rhythm in DPD enzyme activity (Jacobs et al., 2016). Finally, large between-center differences in uracil levels have been observed although the situation has considerably improved recently due to the introduction of external quality control programs (e.g., Asqualab, France) (de With et al., 2022). As was demonstrated in our patient, this test cannot be correctly interpreted when fluoropyrimidines are taken simultaneously or were only recently stopped, due to competitive inhibition of the DPD enzyme (Thomas et al., 2021). It is therefore very important to keep in mind that this phenotyping test should ideally be carried out before any fluoropyrimidine is taken. In case of unexplained fluoropyrimidine toxicity, clinicians should not forget to respect this delay when considering the phenotyping test.

Targeted *DPYD* genotyping for the four most common variants (DPYD*2A, DPYD*13, HapB3 and c.2846A>T (median allele frequency (MAF): 0.28457%; gnomAD v2.1.1, exome data)) is a well-standardized method with a good level of evidence supporting clinical effect and is more cost-effective than more advanced sequencing techniques (Lunenburg et al., 2020). However, as demonstrated in our case, it missed the concurring and rarer variant c.2872A>G (Offer et al., 2014). Moreover, while these variants are the most common causes of 5-FU toxicity in a Caucasian population, this is not the case in other ethnic groups that have been under-represented in large case-control clinical association studies of 5-FU toxicity (White et al., 2021). Because of this, several international expert working groups (Pratt et al., 2024; Garcia-Alfonso et al., 2022), suggest expanding testing to include additional clinically relevant *DPYD* variants, especially in non-European populations and even recommend considering testing for rare variants. Furthermore, with general populations becoming more diverse, testing for only a limited number of variants should be either avoided or accompanied by phenotyping. To date, a combined genotype-phenotype approach prior to initiation of fluoropyrimidine therapy does not appear to necessarily improve toxicity prediction (Etienne-Grimaldi et al., 2017). However, as Next-Generation Sequencing (NGS) technology becomes increasingly cheaper and accessible, more advanced genotyping such as whole genome sequencing and long-read sequencing could potentially offer a more robust solution to detect common as well as rare variants, including compound heterozygosity (Caspar et al., 2021). Although its use in a clinical setting is still limited and requires more research, including the involvement of microRNA, it may substantially improve the prediction of DPYD activity (De Luca et al., 2022; Deac et al., 2021). Nevertheless, *DPYD* phenotyping will persist in its role and may contribute to elucidating the *DPYD* functionalities associated with newly identified unknown variants of which the number will rise with performing whole *DPYD* gene sequencing. Administering therapy based on these unknown variants without understanding their *DPYD* functionality could result in fatal outcomes or reduced treatment efficacy. In retrospect, our patient should have been classified according DPWG as having an activity score of 0.5, prompting the need for additional phenotyping before making any decisions on the final dose (Lunenburg et al., 2020).

Co-existence of two loss-of-function *DPYD* variants has previously been described in oncological patients with confirmed reduced DPD activity and/or observed fluoropyrimidine toxicity (De Falco et al., 2019; Henricks et al., 2017; Baiardi et al., 2023; Johnson et al., 2002; Lau et al., 2023; Shrestha et al., 2018; Detailleur et al., 2021). However, in these cases, the co-existing variants were (moderately) common whereas in our case report the c.2872A>G variant was very rare and occurred only in 0.00199% (5/251,256) of alleles captured in the gnomAD database (v2.1.1, exome data). Furthermore, in previous reports compound heterozygosity was suspected, but *in trans* phasing of the variants could not be confirmed due to the large genomic distance between variants. Confirming that the two inactivating variants occur on the same *DPYD* allele (*in cis*) or on separate *DPYD* alleles (*in trans*) is vital and a particular strength in our case report, as only the latter will lead to non-production of functional DPD and subsequently a poor metabolizer status. Previously, only one patient captured in the 1,000 Genomes database was found to be *in trans* compound heterozygous for two common *DPYD* variants (c.1236G>A and c.2846A>T) (Lunenburg et al., 2018). To our knowledge, the *in trans* compound heterozygosity with the common c.2846A>T variant and the rare c.2872A>G variant identified in our patient has not previously been described. Calculated frequencies for compound heterozygosity for four common *DPYD* variants (DPYD*2A, DPYD*13, c.1236G>A and c.2846A>T) range from 0.0001% to 0.008% (Lunenburg et al., 2018). The calculated frequency of the c.2846A>T/c.2872A>G combination occurring in our patient was 0.000006%. However, despite its rarity the presence of this combination had profound clinical impact on our patient. This highlights the importance of keeping in mind that there is always a small, but by no means ignorable, chance a patient might harbour two *DPYD* variants, resulting in a poor metabolizer status. Furthermore, a recent study of 3,000 patients who underwent both DPD phenotyping and *DPYD* genotyping showed that a considerable amount of interindividual DPD activity could be attributed to rare *DPYD* variants (Larrue et al., 2024). In another study, multivariate analysis showed an increased risk of developing severe fluoropyrimidine toxicity in patients harbouring at least one very rare variant (MAF <0.1%) (De Mattia et al., 2022). Furthermore, inclusion of *DPYD* variants with higher incidence rates in populations of non-European ancestry compared to the European population might improve patient safety and reduce severe fluoropyrimidine-related toxicity (Chan et al., 2024). This issue of rare variants is broader than the *DPYD* gene as it was shown that 10.8% of putatively functional pharmacogenetic variants was considered rare (Ingelman-Sundberg et al., 2018). These results combined with this case report again underscore the importance of integrating rare variants in pharmacogenetic testing, despite the challenges it poses (De Mattia et al., 2024).

In summary, the case described here highlights the importance of preemptive screening for DPD deficiency prior to initiating fluoropyrimidine therapy to avoid severe adverse drug reactions. In addition, it underlines the importance of conducting a DPD phenotyping test, provided it is carried out correctly and in compliance with pre-analytical conditions, as such a test can be more sensitive than a targeted genotyping test, due to the possible presence of rare *DPYD* variants that may affect DPD enzyme activity. The advent of more advanced NGS tests is promising and may circumvent some limitations of both tests. Still, phenotyping will maintain its clinical role in final determination of DPD activity, especially in the case of rare variants.

## Data Availability

The original contributions presented in the study are included in the article/supplementary material, further inquiries can be directed to the corresponding author.
